# Hereditary Hyperferritinemia Cataract Syndrome Mimicking Iron Overload in a Pediatric Patient With Coexisting HFE H63D Homozygosity

**DOI:** 10.1155/crpe/7970734

**Published:** 2026-07-14

**Authors:** Yeşim Yiğit, Hamide Betül Gerik Çelebi

**Affiliations:** ^1^ Department of Pediatric Hematology and Oncology, Balıkesir Atatürk City Hospital, Balıkesir, Türkiye, saglik.gov.tr; ^2^ Department of Medical Genetics, Balıkesir Atatürk City Hospital, Balıkesir, Türkiye, saglik.gov.tr

**Keywords:** cataract, FTL mutation, hereditary hyperferritinemia cataract syndrome, HFE H63D, hyperferritinemia, pediatric

## Abstract

**Background:**

Hereditary hyperferritinemia cataract syndrome (HHCS) is a rare autosomal dominant disorder caused by mutations in the FTL gene, characterized by elevated serum ferritin levels without systemic iron overload and early‐onset cataract. Misinterpretation of hyperferritinemia may lead to unnecessary investigations and treatment.

**Case:**

We report a pediatric case with familial involvement in which hyperferritinemia was incidentally detected in a 9‐year‐old boy. Laboratory evaluation revealed markedly elevated serum ferritin levels with normal transferrin saturation and no evidence of systemic iron overload. Genetic analysis identified a heterozygous c.‐161C > *T* mutation in the FTL gene. In addition, a homozygous HFE c.187C > *G* (p.His63Asp) variant was detected. Family screening revealed similar biochemical and genetic findings in multiple relatives, several of whom had bilateral cataracts.

**Conclusion:**

This case underscores the importance of considering HHCS in pediatric patients presenting with hyperferritinemia and normal transferrin saturation. The coexistence of FTL mutation and HFE H63D homozygosity appears to be incidental and should be interpreted cautiously. Recognition of this condition is essential to prevent unnecessary investigations and inappropriate treatment.

## 1. Introduction

Hereditary hyperferritinemia cataract syndrome (HHCS) is a rare autosomal dominant disorder caused by mutations in the iron‐responsive element of the ferritin light chain (FTL) gene. It is characterized by persistently elevated serum ferritin levels in the absence of systemic iron overload and the development of early‐onset bilateral cataract [1, 2]. Mutations in the FTL gene disrupt the interaction between iron regulatory proteins and the iron‐responsive element, resulting in increased L‐ferritin synthesis independent of body iron stores [2, 3].

HHCS is frequently underrecognized and may be misinterpreted as hereditary hemochromatosis, particularly in patients presenting with isolated hyperferritinemia [4]. This misinterpretation may lead to unnecessary diagnostic procedures and inappropriate treatments such as phlebotomy. A key distinguishing feature is normal transferrin saturation, which helps differentiate HHCS from iron overload disorders [4, 5].

Mutations in the HFE gene, particularly the H63D variant, are associated with hereditary hemochromatosis; however, the clinical penetrance of H63D is low, and its contribution to iron overload is generally limited [6, 7]. Coexistence of FTL mutations and HFE variants has rarely been reported and may introduce diagnostic uncertainty [8].

Here, we present a pediatric case with familial findings of HHCS associated with hyperferritinemia and cataract, in whom coexisting HFE H63D homozygosity was identified. This report highlights an important diagnostic pitfall in the evaluation of hyperferritinemia and emphasizes the importance of integrating clinical, biochemical, and genetic findings to avoid misdiagnosis and unnecessary treatment.

## 2. Case Presentation

A 9‐year‐old boy was referred to our clinic after hyperferritinemia was incidentally detected during routine evaluation (serum ferritin: 1500–1841 ng/mL). He had no significant past medical history and no history of blood transfusion. Family history was notable for bilateral cataracts and Type 1 diabetes mellitus in the father. There was no history of liver disease, arthritis, or consanguinity.

On physical examination, the patient’s growth parameters were within normal percentiles (height: 134 cm, 60th percentile; weight: 33 kg, 75th percentile). Vital signs were normal, and no hepatosplenomegaly was detected. Echocardiography and abdominal ultrasonography were unremarkable.

Given persistent hyperferritinemia, an ophthalmologic examination was performed, which showed bilateral cataracts.

Ophthalmologic evaluation further demonstrated bilateral lens opacities consistent with HHCS. The father had previously undergone cataract surgery, whereas visual acuity was preserved in the pediatric patients, and no surgical intervention was required at the time of evaluation.

Laboratory evaluation demonstrated markedly elevated serum ferritin (1841 ng/mL) with normal transferrin saturation (39%). The hemoglobin level was within normal limits. There was no biochemical or radiological evidence of iron overload.

Given the discrepancy between elevated ferritin levels and normal transferrin saturation, genetic evaluation was performed. Analysis identified a heterozygous c.‐161C > *T* mutation in the FTL gene, consistent with HHCS. In addition, a homozygous HFE c.187C > *G* (p.His63Asp) variant was detected.

Family screening was subsequently conducted. The father had markedly elevated ferritin levels (2136 ng/mL), bilateral cataracts, and carried both the heterozygous FTL mutation and homozygous HFE H63D variant. A 7‐year‐old sibling also demonstrated hyperferritinemia (2025 ng/mL), bilateral cataracts, and similar genetic findings. A 1‐year‐old sibling had elevated ferritin levels (2332 ng/mL) and carried the same genetic mutations but had not yet developed cataract. The mother had normal ferritin levels and no identified mutations.

Clinical and laboratory findings of all family members are summarized in Table 1.

**TABLE 1:** Clinical and laboratory findings of family members.

Individual	Age	Hemoglobin (g/dL)	Ferritin (ng/mL)	Transferrin saturation (%)	Cataract	HFE mutation	FTL mutation
Mother	32	11.0	28	12%	No	—	—
Father	34	15.2	2136	29%	Bilateral	Homozygous c.187C > *G* (p.His63Asp)	Heterozygous c.‐161C > *T*
Patient	9	13.0	1841	39%	Bilateral	Homozygous c.187C > *G* (p.His63Asp)	Heterozygous c.‐161C > *T*
Sibling 1	7	11.0	2025	23%	Bilateral	Heterozygous c.187C > *G* (p.His63Asp)	Heterozygous c.‐161C > *T*
Sibling 2	1	11.2	2332	5%	No	Homozygous c.187C > *G* (p.His63Asp)	Heterozygous c.‐161C > *T*

*Note:* FTL: ferritin light chain gene; HFE: gene associated with hereditary hemochromatosis.

Based on the presence of persistently elevated ferritin levels, normal transferrin saturation, early‐onset cataract, and identification of an FTL mutation, the diagnosis of HHCS was established. The coexisting HFE H63D homozygosity was considered an incidental finding and was not thought to contribute significantly to the clinical phenotype.

## 3. Discussion

HHCS is characterized by elevated serum ferritin levels without systemic iron overload and early‐onset bilateral cataract [1, 2]. The underlying mechanism involves mutations in the iron‐responsive element of the FTL gene, leading to dysregulated ferritin synthesis independent of body iron stores [2, 3].

The FTL c.‐161C > *T* variant has been previously reported in association with HHCS in several studies, supporting its pathogenic role through disruption of the iron‐responsive element.

In clinical practice, HHCS is frequently misinterpreted as hereditary hemochromatosis, particularly in patients presenting with isolated hyperferritinemia [4]. This may result in unnecessary diagnostic procedures and inappropriate therapeutic interventions such as phlebotomy. In our case, markedly elevated ferritin levels in the presence of normal transferrin saturation were key findings supporting HHCS rather than iron overload.

An additional aspect in this patient was the coexistence of the homozygous HFE H63D mutation. Compared to the C282Y mutation, which is strongly associated with clinically significant iron overload, the H63D variant is considered a low‐penetrance allele and rarely leads to overt hemochromatosis, particularly in pediatric populations. Its clinical expression is often age dependent and may manifest later in adulthood.

The absence of elevated transferrin saturation and lack of organ involvement in this case support HHCS as the primary cause of hyperferritinemia, and the HFE variant is most likely an incidental finding. In addition, the lack of correlation between the HFE genotype and ferritin levels within this family further reinforces its limited clinical significance.

The coexistence of FTL and HFE mutations has rarely been reported [6]. This combination may create diagnostic confusion, particularly in pediatric patients, where hyperferritinemia often triggers extensive evaluation. Our findings highlight the importance of careful interpretation of iron parameters and the need for genetic testing when ferritin levels are elevated in the absence of clear evidence of iron overload.

Another important aspect of HHCS is the pathophysiology of cataract formation. Increased L‐ferritin synthesis leads to its accumulation within the ocular lens, where limited protein turnover and high protein concentration promote ferritin aggregation and crystallization, ultimately resulting in lens opacity [2, 7]. This mechanism explains the early development of cataracts in affected individuals.

Our findings have several clinical implications. First, HHCS should be considered in the differential diagnosis of hyperferritinemia, particularly when transferrin saturation is normal. Second, family screening is essential for identifying affected individuals and preventing unnecessary interventions. Finally, genetic confirmation is crucial for accurate diagnosis and appropriate management.

In this case, we report the coexistence of an FTL mutation and HFE H63D homozygosity. These findings emphasize that not all cases of hyperferritinemia indicate iron overload and highlight the importance of integrating clinical, laboratory, and genetic data to ensure accurate diagnosis and avoid unnecessary interventions.

## Funding

No funding was received for this manuscript.

## Ethics Statement

Ethics committee approval was not required for this case report according to institutional policies.

## Consent

Written informed consent was obtained from the patients’ parents.

## Conflicts of Interest

The authors declare no conflicts of interest.

## Data Availability

The data that support the findings of this study are available on request from the corresponding author. The data are not publicly available due to privacy or ethical restrictions.
